# Longitudinal analytical approaches to genetic data

**DOI:** 10.1186/s12863-015-0312-y

**Published:** 2016-02-03

**Authors:** Yen-Feng Chiu, Anne E. Justice, Phillip E. Melton

**Affiliations:** Division of Biostatistics and Bioinformatics, Institute of Population Health Sciences, National Health Research Institutes, Miaoli, Taiwan, ROC; Department of Epidemiology, University of North Carolina, Chapel Hill, NC 27514 USA; Centre for Genetic Origins of Health and Disease, University of Western Australia, Perth, WA Australia

## Abstract

**Background:**

Longitudinal phenotypic data provides a rich potential resource for genetic studies which may allow for greater understanding of variants and their covariates over time. Herein, we review 3 longitudinal analytical approaches from the Genetic Analysis Workshop 19 (GAW19). These contributions investigated both genome-wide association (GWA) and whole genome sequence (WGS) data from odd numbered chromosomes on up to 4 time points for blood pressure–related phenotypes. The statistical models used included generalized estimating equations (GEEs), latent class growth modeling (LCGM), linear mixed-effect (LME), and variance components (VC). The goal of these analyses was to test statistical approaches that use repeat measurements to increase genetic signal for variant identification.

**Results:**

Two analytical methods were applied to the GAW19: GWA using real phenotypic data, and one approach to WGS using 200 simulated replicates. The first GWA approach applied a GEE-based model to identify gene-based associations with 4 derived hypertension phenotypes. This GEE model identified 1 significant locus, *GRM7*, which passed multiple test corrections for 2 hypertension-derived traits. The second GWA approach employed the LME to estimate genetic associations with systolic blood pressure (SBP) change trajectories identified using LCGM. This LCGM method identified 5 SBP trajectories and association analyses identified a genome-wide significant locus, near *ATOX1* (*p* = 1.0E^−8^). Finally, a third VC-based model using WGS and simulated SBP phenotypes that constrained the β coefficient for a genetic variant across each time point was calculated and compared to an unconstrained approach. This constrained VC approach demonstrated increased power for WGS variants of moderate effect, but when larger genetic effects were present, averaging across time points was as effective.

**Conclusion:**

In this paper, we summarize 3 GAW19 contributions applying novel statistical methods and testing previously proposed techniques under alternative conditions for longitudinal genetic association. We conclude that these approaches when appropriately applied have the potential to: (a) increase statistical power; (b) decrease trait heterogeneity and standard error; (c) decrease computational burden in WGS; and (d) have the potential to identify genetic variants influencing subphenotypes important for understanding disease progression.

## Background

Analysis of longitudinal measurements in genetic epidemiology provides a methodological strategy for the understanding of changes affecting long-term averages and changes in complex disease phenotypes over time. The design of these longitudinal studies may provide additional phenotypic information regarding age of onset, allowing for more precise definition of complex disease status. Inclusion of repeat measurements potentially allows for an increased understanding of the trajectory of traits and disease progression. Finally, longitudinal studies permit the perspective measurement of time-varying covariates and potential interactions that are not accounted for in traditional genetic studies focused on an individual time point [1].

In addition to these advantages, there are challenges in the appropriate statistical methodology and interpretation of longitudinal data. Repeat measurements from the same subject are often correlated and those statistical models that assume independent measurements are inappropriate. In family-based genetic studies, correlation among family members also needs to be considered [2]. The inclusion of collapsed summary statistics (ie, average of repeat measurements, slope) or repeat measurements for each individual needs to be carefully considered. Finally, the computational routines utilized need to be properly evaluated, as advanced statistical methods such as generalized estimating equations (GEEs) and linear mixed-effect (LME) models that account for the addition of pedigree structure may not be scalable to large genetic data sets.

A number of recent genetic epidemiological studies have used longitudinal measurements in order to take advantage of repeat measurements of time-varying variables [3–6]. These studies have focused on the application of current high-density genotype and simulated data to assess the statistical power and accuracy of including repeat measurements in genetic studies. In addition, participants in 3 previous Genetic Analysis Workshops (GAWs)—GAW13, GAW16, and GAW18—have sought to develop statistical approaches to observed and simulated repeat measurements [2, 7–9]. All these previous GAW groups presented a heterogeneous range of statistical methods for longitudinal analysis. The GAW13 group investigated phenotypic and genetic data from the Framingham Heart Study (FHS) families and categorized 13 contributions into 2 basic approaches: (a) 2-step modeling, where repeat measurements were collapsed to 1 summary statistic per individual (ie, average, slope) and this statistic was used in standard univariate statistical models; and (b) joint modeling, where model parameters were estimated simultaneously. For summary statistic collapsing, they found that the mean-type statistic provided greater statistical power than the slope-type statistic [8]. The GAW16 group also investigated FHS family data focusing on single nucleotide polymorphism (SNP) genotypes and cardiovascular-related phenotypes. They did not divide their approaches into hierarchical subdivisions, but concluded that the precision of the genetic effects on the phenotype is improved with the incorporation of longitudinal data [9].

Two GAW18 groups investigated longitudinal measurements, with one group focused on methods using genome-wide association (GWA) SNPs and a second group focused on whole genome sequence (WGS) data or a combination of GWA and WGS. Both of these groups used blood pressure phenotypes from the Type 2 Diabetes Genetic Exploration by Next-Generation Sequencing in Ethnic Samples (T2D-GENES) consortium. In addition, for GAW18, there was a limited sample of unrelated individuals [2, 7]. The GAW18 GWA group focused on 8 longitudinal methods and their comparison with univariate association models. They found that missing data and limited sample size were the most common challenges and that this led to little concordance of the findings across groups [7]. The GAW18 WGS and GWA group categorized 8 contributions into 2 broad groups based on handling of dependence structures: (a) LME, where random effects are used to capture dependence structures; and (b) variance component (VC) models, where dependence structures are constructed based on multiple components of VC matrices for the multivariate Gaussian response. In this group, there was little consensus on overall findings, a result of the heterogeneous nature of the contributions, but they came to 3 conclusions: (a) statistical power can be gained with longitudinal measurements; (b) inclusion of family structure allows for more accurate genotyping and detection of rare variants; and (c) fitting LME and VC models for repeat measurements presents computational challenges [2]. The common nature of all previous GAW longitudinal groups was the variety of statistical procedures applied, which often did not allow for direct comparison of results across the different models. This heterogeneous nature of statistical approaches for longitudinal data in previous GAWs, with no standard procedure used for comparison, is a potential reason for the lack of utilization of these proposed methods outside of statistical genetic workshops.

In this paper, we summarize 3 GAW19 contributions (Table 1) that are focused on the development of statistical methods using repeat measurement and either GWA [10, 11] or WGS [12]. Similar to previous GAWs, investigators in this group applied a variety of statistical approaches and strategies to deal with the advantages and challenges incorporating longitudinal measurements mentioned above. The focus of our GAW19 working group was on the use of statistical procedures to identify either common (GWA) or rare (WGS) variants associated with phenotypic variation using repeat measurements. All of these discussed contributions used pedigree-based information, as only the family-based data contained repeat measurements. The 2 GWA contributions analyzed the real GAW19 data [10, 11], while the WGS contribution used the simulated GAW19 data set [12] with prior knowledge of the answers.

**Table 1:** Longitudinal GAW19 statistical models, data set, and software used by this group

Contribution	Phenotype	Covariates	Genetic data	Data Set	Model(s)	Longitudinal correlation	Software
Chiu et al. [10]	Hypertension status (Baseline, Longitudinal, Ever, Progression)	None	GWA chromosome 3	Real	multipoint LD mapping using GEE	Independent working correlation	Author’s software in FORTRAN
Justice et al. [11]	SBP adjusted for BP medication	Age, sex, PCs (1–4), (smoking nonsignificant)	GWA	Real	LCGM (phenotype) and VCs within mixed model (association)	Unstructured covariance	SAS (LCGM) MMAP (association)
Melton et al. [12]	SBP	Age, sex, and smoking	WGS	Simulated	VC	Correlation between SBP responses	SOLAR

*BP* blood pressure, *GEE* generalized estimating equations, *GWA* genome-wide association, *LCGM* latent class growth modeling, *LD* linkage disequilibrium, *MMAP* mixed models analysis for pedigrees, *PC* principal components, *SAS* statistical analysis system, *SBP* systolic blood pressure, *SOLAR* sequential oligogenic linkage analysis routines, *VC* variance-components, *WGS* whole-genome sequence

## Methods

### GAW19 data

GAW19 data utilized a format similar to that used in GAW18 [13, 14], with corrections on a few individuals’ phenotypes and the inclusion of transcriptomic probes from the microarray. It included a real and a simulated data set of 200 phenotype replicates. The real data set was provided by T2D-GENES and included information from 20 Mexican American families, with WGS for 464 selected individuals and approximately 500,000 GWA SNPs for 959 pedigree members. Real phenotypes included sex, along with repeat measurements from 4 examinations for systolic (SBP) and diastolic blood pressure (DBP), age, year of examination, antihypertensive medication use, and current tobacco smoking status. The average follow-up interval between 2 visits was 5.27 years. The missing frequency of hypertension status/blood pressure at the 4 visits was 17 %, 40 %, 40 %, and 76 %, respectively.

Simulated WGS data, cleaned of Mendelian errors, were provided for 959 individuals (464 directly sequenced and the remaining imputed) for 8,348,674 variants from odd-numbered autosomes and did not include structural variants. The 200 simulated replicates included phenotypic measurements from 3 time points generated using the same pedigree structures and the same 849 individuals who had both phenotype and imputed sequence data in the real data set. For individuals not examined at all-time points in the real data set, missing ages at exam were filled in by adding or subtracting 3.9 years between exams 1 and 2 and/or 6.9 years between exams 1 and 3. Information regarding SBP, DBP, hypertension diagnosis, medication use, and tobacco smoking were generated for each simulation replicate.

### GAW19 approaches

#### Genome-wide association

In the first GWA approach, Chiu et al. [10] developed a multipoint linkage disequilibrium (LD) mapping method to localize disease susceptibility loci using chromosome 3 GWA SNPs (see Table 1). Their model accounted for correlations between subjects in families, between markers, and repeat measurements within subjects, simultaneously using the GEE approach [15, 16]. An independent working correlation matrix was assumed in GEEs. Four different ways of defining hypertension phenotypes were evaluated: (a) ever having hypertension *(Ever)*; (b) incidence event with status changed from unaffected to affected *(Progression)*; (c) first available visit as baseline only *(Baseline)*; and (d) all available time points *(Longitudinal)*. They compared the estimates of the disease locus positions, their standard errors, the genetic effect estimate at the disease loci, and their significance for the 4 phenotypes to examine the efficiency gained from using repeatedly measured phenotypes. In this approach, genotypes are treated as random. Given the phenotype, these missing GWA SNPs are presumably independent of the phenotype, making missing completely at random (MCAR) a legitimate assumption.

In the second GWA approach, Justice et al. [11] aimed to identify susceptibility loci associated with SBP change trajectories. All subjects with at least 2 measurements were included, assuming missing data were MCAR. First, the structural equation modeling (SEM) implemented in Mplus v7.11 [17] was used to identify covariates in estimating the SBP change trajectories while taking into account potential genetic effects using SNPs previously identified for SBP, DBP, and pulse pressure (PP). GEE was used within the SEM framework to account for the correlations within an individual and within a pedigree. Significant covariates in the SEM were included to account for their impact on an individual’s trajectory through a semiparametric latent class growth modeling (LCGM). Identification of SBP trajectories was conducted assuming a censored normal distribution using multivariate mixture models implemented in PROC TRAJ in SAS version 9.2 (Cary, NC, USA). This model assumes that given class membership, the repeated measurements for the *i*^th^ individual are independent. Each LCGM trajectory group was ranked based on health risk, defined as the average number of cumulative years as hypertensive (ie, members of group 1 exhibiting the fewest number of hypertensive years and group 5 the greatest). Pairwise GWA analyses between groups, with group 1 as the referent group, were conducted using the MMAP (mixed models analysis for pedigrees) computing package [18]. MMAP estimates VCs within the LMM framework to account for relatedness between individuals assuming an unstructured covariance. An additive genetic model was assumed, and the first 4 principal components (PCs) as fixed effects were included to control for population structure. All available SNPs on odd-numbered chromosomes and with a minor allele frequency of less than 1 % were analyzed. Additionally, to make use of the entire data set, the rank ordered groups of trajectories were treated as a continuous trait in the GWA.

#### Whole genome sequence

In the third approach, Melton et al. [12] conducted multivariate association for the WGS within the VC framework to test for main effects on SBP measurements across 3 time points from 200 simulated phenotype replicates. This technique built upon a constrained maximum likelihood approach, which takes into account all repeat measurements of a phenotype in families. This method investigated the effect of a variant on the mean trait values of all measured longitudinal phenotypes by constraining the displacement in trait means with each copy of the minor allele to be equal. The genetic and environmental correlations are modeled through random effects allowing for correlations between all measurements. Comparisons were made among 3 statistical approaches: (a) constrained, where the β coefficient of a variant or gene region on the mean trait value was constrained to be equal across all measurements; (b) unconstrained, where the variant or gene region β coefficient was estimated separately for each time point; and (c) the average SBP measurement from 3 time points. They first conducted a univariate approach of the average of SBP measurements from 3 time points using 9 variants from the GAW19 answers, and 20 randomly ascertained variants as negative controls. Then, both constrained and unconstrained multivariate association were conducted for the same variants. Finally, a gene-based test was performed for 2 regions, the *MAP4* region on chromosome 3 and a randomly ascertained equivalent region on chromosome 1. The gene-based test applies gene-specific relationship matrices to determine the proportion of the trait’s variance explained by an individual gene as a result of the departure of its localized empirical kinship estimate from the pedigree-derived theoretical kinship estimates. All analyses included the covariates baseline age, sex, their interactions, smoking status, and were performed using the computer package SOLAR (Sequential Oligogenic Linkage Analysis Routines) [19].

## Results

### Genome-wide association

In the first GWA approach, Chiu et al. [10] used multipoint LD with GEE to conduct gene-based association tests between SNPs within 1095 genes on chromosome 3 and 4 derived phenotypes of hypertension status: (a) *Ever*; (b) *Progression*; (c) *Baseline*; and (d) *Longitudinal*. Of 1095 genes tested, 119 *(Ever)*, 79 *(Progression)*, and 42 *(Longitudinal)* were significantly associated (*P* <4.57 × 10^−5^), using a Bonferroni multiple correction for number of genes, with hypertension status. A total of 49 genes were identified in association with *Baseline* hypertension. Several gene regions identified in this study were near previously implicated hypertension genes (eg, *GRM7*, *SLC4A7*, *ADAMTS9*). Also, the results of the *Ever* and *Progression* phenotypes were very similar, with 21 overlapping significant genes and similar standard errors. There was 1 overlapping locus, *GRM7*, which passed multiple test corrections for both *Baseline* and *Longitudinal*. Additionally, of those gene regions with nominal significance (*p* <0.05) for both *Longitudinal* and *Baseline* phenotypes, 64 % of these regions exhibited lower standard error for the *Longitudinal* phenotype as compared to *Baseline* [10].

In the second GWA approach, Justice et al. [11] found that sex, age, and PCs were important covariates for SNP associations with temporal changes in SBP. After adjusting for sex at each time point, 5 distinct SBP change trajectory classes were identified in the LCGM modeled against age. These 5 classes were ranked based on perceived health risk by average number of observed cumulative years as hypertensive, class 1 with the lowest number of cumulative years as hypertensive and class 5 with the highest number of cumulative years. Association analyses identified 8 loci (index SNP ± 500 kb) that reached suggestive significance (*p* <1.6E-6) in 1 or more pairwise-association tests, and including one that reached genome-wide significance (*p* <1.3E-7) near the *ATOX1* gene (rs17112252, *p* = 1E-8) for the pairwise association analysis of trajectory group 2. The greatest number of suggestive loci (4) were identified in trajectory class 5, the class with the greatest perceived health risk and the smallest sample size (*N* = 136), including rs4756864 (p = 1.7E-7) within a previously implicated SBP associated region [11, 20].

### Whole genome sequence

In the third approach for WGS, Melton et al. [12] successfully identified the simulated association of the *MAP4* variants 3_48040283 and 3_47957996 as being genome-wide significant (*p* value <5.0E-9) in 99.5 % of the 200 replicates for the unconstrained and 100 % of the replicates for the constrained method. For the remaining 7 variants tested, there was some variation across the models, but overall the constrained approach showed increased power. Although when larger genetic effects were present, collapsing, using the average across measures, was equally effective. For the gene-based test, averaging all repeat measurements displayed the greatest power in detecting the simulated true-positive effects for the *MAP4* region. The gene-based test using only baseline SBP had greater power to detect true-positive effects than did either the constrained or unconstrained models, but this was likely because the authors used all available variants in each region tested rather than prioritizing variants based on functional annotation [21]. Overall, the gene-based approach was less powerful at detecting significant associations in the *MAP4* region as compared to the individual variant association tests.

## Discussion

A direct comparison of results between each contribution summarized herein is made difficult by the variability in phenotype, genotype, and analytical method implemented (see Table 1). However, there are some common strengths and potential limitations of these statistical approaches that can be highlighted. Additionally, these papers bring to light some important avenues for further exploration in the genetic association of longitudinally assessed phenotypes.

### Challenges for incorporating longitudinal data

#### Number of time points

Given the stochastic changes in observations across age/time, if SBP or hypertension status is measured at only a single time point, the genetic effect may be over- or underestimated. Over time, and as more measurements are collected, we may obtain better estimates and converge to the true genetic effect. However, the present studies are limited by the number of available time/age points at which measures were taken. Both Chiu et al. [10] and Justice et al. [11] used the real GAW19 data set, which included up to 4 measures, but was subject to missing data. Melton et al. [12] used the simulated GAW19 phenotypes with 3 available time points, but no missing information. The small number of time points may be more of a limiting factor for Justice et al. [11]. Their SBP change trajectories were modeled against 4 measures of age and, therefore, limited to the quadratic term. It is likely that with additional time/age points, predicted SBP trajectories [11] would be changed and genetic effect estimates would be improved [10, 12].

#### Availability of informative covariates

As mentioned above, only a select number of covariates were available for inclusion in these analyses (age, sex, smoking, medication use). There are other potential variables that may be of interest when adjusting SBP and hypertension status, and that may be of particular concern for longitudinal data. For example, behavioral recommendations that accompany medical prescriptions could be important mediators or effect modifiers for the genetic influence of changes in blood pressure between visits at the same scale as medication use (eg, physical activity, diet) [22, 23]. However, behavioral covariates other than smoking were not included for GAW19.

### Strengths identified

#### Decreased computational and analyst burden

As noted in the GAW18 longitudinal WGS and GWA summary paper, computational burden, total analysis time, and analyst burden may be limiting factors, especially for WGS [2]. However, these 3 GAW19 contributions attempted to address some issues imposed by conducting genetic analysis on repeat measurements. All 3 groups applied a form of collapsing, or used a derived variable by collapsing repeated measurements to a single measure or group variable (eg, the average of all measures, trajectory class, hypertension progression). Using WGS, Melton et al. [12] showed that a univariate average across all time points not only increased power to detect genetic effects, but by collapsing the repeat measurements into a single phenotype, allowed for utilization of the software program, SOLAR [19], which reduced analyst burden and computational time. Although the evaluated multipoint LD method implemented by Chiu et al. [10] does require sophisticated analytical and programming skills, multiple repeat measurements helped improve efficiency of the estimates. These phenotypic collapsing techniques can also be analyzed using alternative GWA approaches. Justice et al. [11] used group-based trajectory analysis to identify subphenotypes of SBP change for use in GWA using widely available software [11]. This approach also reduces computational time, and analyst burden; however, no direct comparison was performed with cross-sectional GWA of SBP.

#### Incorporation of correlation between time points, which can increase power

One of the greatest possible benefits for longitudinal data is in the use of correlations between repeat measurements within subjects, which increases power to detect genetic effects by decreasing trait heterogeneity. Chiu et al. [10] and Melton et al. [12] both leveraged these correlations in their approaches, analyzing hypertension across time using multipoint [10] and maximum likelihood for multivariate association [12], respectively. Melton et al. [12] showed an increase in power for longitudinally measured phenotypes over a single measure. Alternatively, the LCGMs used by Justice et al. [11] are based on the assumption that given class membership, the observations within a person are independent. The correlation among repeated measures, according to these models, is captured completely by the trajectory class of each individual [11]. We cannot comment directly on the possible improvement in power for Chiu et al. [10] or Justice et al. [11] as both studies applied these methods to real data and therefore the “true” associations are unknown. However, generalization of previously implicated hypertension *(GRM7, SLC4A7, ADAMTS9)* and SBP-associated loci *(PLEKHA7)* from cross-sectional analyses with much larger sample sizes, provide strong evidence that these statistical models are robust. Future work will be required to definitively show increased power using either method.

#### Identify unique trajectories of disease-risk progression

Recent studies that have partitioned heterogeneous phenotypes into meaningful subphenotypes have proven useful for the identification of novel genetic susceptibilities and allowed for the estimation of previously missing heritability in complex disease traits (eg, cancer, autism, schizophrenia) [24–27]. These studies suggest that methodological innovations that identify homogenous subphenotypes may be useful for characterizing the genetic influence of longitudinal phenotypic change and disease progression [6]. Justice et al. [11] chose to use a LCGM trajectory method to identify underlying classes of SBP changes. In other words, this research group was primarily interested in identifying genetic risk variants that increase susceptibility to patterns of SBP change across age that may provide additional information on hypertension risk and prognosis. Similar approaches have been proposed elsewhere and validated using simulated continuous traits [6]. Similarly, Chiu et al. [10] defined important subphenotypes of incident hypertension that are shown to improve effect estimation and may represent important subphenotypes for disease progression [10].

#### Evidence of improvement of effect estimation over cross-sectional analysis

Chiu et al. [10] showed that using longitudinal assessments of incident hypertension (*Ever*, *Progression*, and *Longitudinal*) decreased the standard error of effect estimates over *Baseline*-only phenotype analyses. This method may prove useful for analyzing other incident diseases across time (eg, diabetes, stroke, coronary heart disease).

#### Improve ability to detect rare variants with moderate to large genetic effects, even with minimal collapsed phenotypes (average across measurements)

WGS poses an increased challenge for longitudinal data, because of rare-variant identification and the computational burden of analyzing millions of markers simultaneously using complex, multilevel models. Family-based studies are useful for conducting rare-variant identification because of their ability to distinguish true variants from sequencing errors, reduce genetic heterogeneity, and increase the number of allelic copies. Therefore, the GAW19 family data set is ideal for improving statistical models incorporating repeat measurements using WGS. Additionally, Melton et al. [12] were able to improve the ability to detect rare variant associations by using minimal collapsing methods, thus decreasing computational burden and time.

## Conclusions

In this paper, we summarize 3 GAW19 contributions applying novel statistical methods and testing of previously proposed techniques under alternative conditions for longitudinal genetic association. Although these statistical techniques varied, each evaluated the potential benefits of using phenotype collapsing and noncollapsing approaches to analyze longitudinal phenotypes. These longitudinal approaches may: (a) increase power to detect genetic effects; (b) decrease trait heterogeneity and standard error of effect estimates; (c) decrease computational burden for WGS; and (d) have the potential to identify subphenotypes important for understanding disease progression. Disease progression is a complicated process; however, genetic variants and biological pathways will offer new means to identify disease susceptibility, predict progression, and highlight effective public health interventions.
